# Primary Malignant Melanoma of the Small Intestine Presenting with Peritonitis: A Case Report

**DOI:** 10.30699/IJP.2023.1996329.3100

**Published:** 2023-12-29

**Authors:** Alireza Khooei, Sahar Seifnia, Amin Dalili, Hossein Bavandi, Saeid Dehghan Nezhad, Motahare Ebrahimnejad

**Affiliations:** 1 Department of Pathology, Faculty of Medicine, Mashhad University of Medical Sciences, Mashhad, Iran; 2 Surgical Oncology Research Center, Faculty of Medicine, Mashhad University of Medical Sciences, Mashhad, Iran

**Keywords:** Malignant melanoma, Peritonitis, Small intestine

## Abstract

Malignant melanoma of the small intestine is mostly a metastatic tumor of other primary lesions, especially of skin origin. Primary malignant melanoma of the small intestine is very uncommon. The clinical presentation is usually nonspecific, thus leading to late diagnosis.

We report a 42-year-old man who presented to the emergency department of Imam-Reza Hospital with symptoms and signs of peritonitis and was a candidate for emergency laparotomy and enterectomy. The medical and family history were unremarkable. A bulky mass was seen 190 cm away from the Treitz band, and a diagnosis of malignant melanoma was confirmed by histologic and immunohistochemical study. Further clinical examination revealed no primary tumor elsewhere, so the diagnosis of primary small intestinal melanoma was concluded.

Although metastatic malignant melanoma in the GI tract is common, the primary one is a very rare entity. The diagnosis could be challenging because a thorough investigation is needed to rule out the possible initial origin.

## Introduction

Primary malignant melanoma accounts for 1-3% of all malignant tumors of the gastrointestinal (GI) system (1). Most cases of malignant melanomas in the GI tract are metastatic from primary mucocutaneous tumors (2). Differentiation of the metastatic melanoma of the GI tract from the primary ones can be difficult (3). Histological misdiagnosis is frequent because melanin pigment is present in only 30% of patients. Therefore, immunohistochemical panels like anti-S-100 protein, HMB-45, and Melan-A would be helpful (4). Sometimes, low-pigment or amelanotic melanomas create a significant diagnostic problem and must be distinguished from other common intestinal tumors such as lymphoma, poorly differentiated carcinoma, sarcomas, Gastrointestinal Stromal tumors, neuroendocrine tumors, and other tumors (5). Compared to non-digestive cases, gastrointestinal melanomas behave more aggressively, and the prognosis is poor. The average survival rate over five years is less than 10% (2).

## Case Presentation

**Fig. 1:**
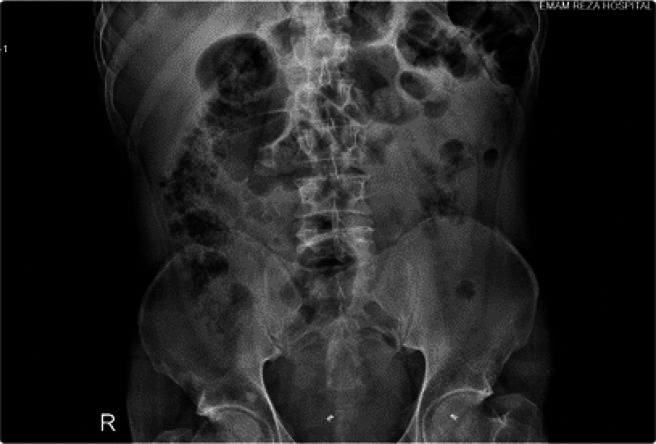
Plain abdominal X-ray showing distention at the proximal and air-free level at the distal loop

**Fig. 2:**
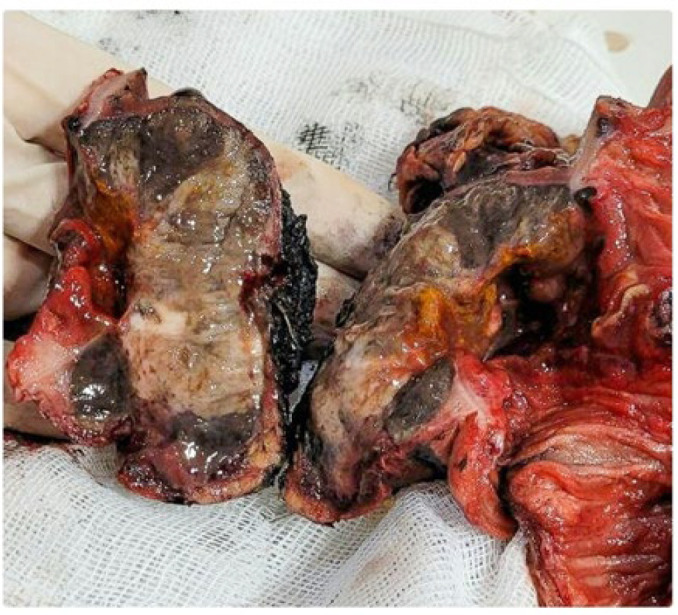
Emergent laparotomy showing a bulky tumor in the small intestine

**Fig. 3:**
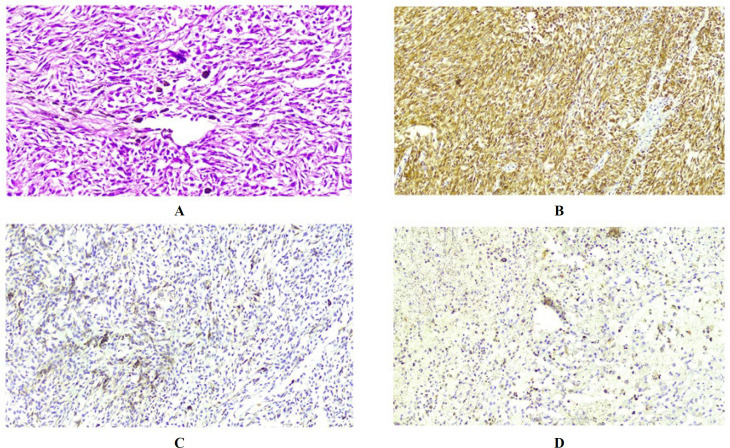
Hematoxylin-eosin section (H&E) and immunohistochemical study of malignant melanoma. H&E staining showing malignant spindled-shaped and epithelioid cells with high cellularity (A). An immunohistochemical study showing strong positive immunoreactivity for HMB-45 (B) and a partial positive reaction for Melan-A (C) and S-100 (D)

Here we report a 42-year-old man with a 4-month history of vague slight abdominal pain who was suffering from acute abdominal pain and persistent vomiting since the day before referral to the emergency department of Imam-Reza Hospital. The medical history was unremarkable. Peritonitis was presented as a primary clinical diagnosis based on the clinical examination. The abdominal ultrasound conducted in the emergency department revealed “segmental intestinal dilation with wall thickening”; a plain abdominal X-ray revealed dilated proximal bowel loops and air-free level in the distal part (Figure 1). Routine laboratory tests showed leukocytosis. Based on radiographic and clinical findings, an emergency laparotomy was done. The intraoperative finding was a bulky greyish tumor (7 x 6 cm) of the small bowel with focal ulceration and necrosis, which extended up to the serosal layer (Figure 2). The tumor was located 190 cm away from the Treitz band; a related loop of bowel closely adhered to the sigmoid. A surgical resection of 30 cm from the small intestine, which included a tumor, as well as the removal of lymph nodes around the intestine and sigmoid due to adhesion to the intestine, was performed. Histological findings showed a proliferation of spindled-shaped and epithelioid cells with irregular nuclear borders, prominent nucleoli, and a few cells with intracytoplasmic melanin pigment (Figure 3).

**Fig. 4:**
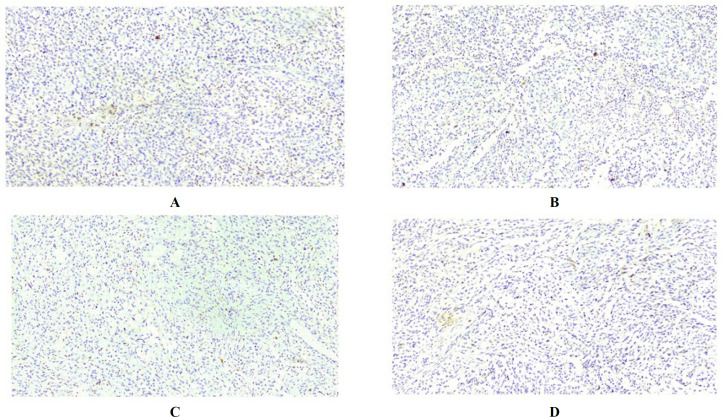
Immunohistochemical study to rule out other mimics. Immunohistochemical stains showing negative immunoreactivity for CD117 (A), DOG1 (B), chromogranin (C), and cytokeratin (D)

Additionally, there were areas of necrosis and acute inflammation in the wall of the small intestine. The immunohistochemical study revealed strong positivity for HMB-45 in nearly all the tumoral cells with focal positivity of Melan-A and S-100 protein (Figure 3). CD117, DOG1, chromogranin, and cytokeratin was negative in the tumoral cells, which helped to rule out the possibility of a GIST, neuroendocrine tumor, and poorly differentiated carcinomas (Figure 4). Based on morphological and immunohistochemical findings, the tumor was consistent with malignant melanoma. All the regional lymph nodes were negative for metastasis; all margins of the surgical resection were free, and no implantation was identified in the peritoneum and visceral serosa. Through investigation no primary source for malignant melanoma outside the small bowel was found.

## Discussion

Malignant melanoma is a common malignant tumor, accounting for about 2% of the cancers (6). Primary non-cutaneous melanoma is an uncommon condition, often occurring in the mucosa of the eye or leptomeninges (7). Malignant melanoma in the GI tract is rare and predominantly metastatic from cutaneous primary origins (8). “Primary mucosal melanoma can arise at any site within the GI mucosa, but it is most common in anorectal (anal canal,

31.4%; rectum, 22.2%) and oropharyngeal (32.8%) regions, whereas esophagus (5.9%), stomach (2.7%), small intestine (2.3%), gallbladder (1.4%), and large intestine (0.9%) are infrequent sites of origin” (2). Different theories have described the origin of the small intestinal melanoma. Although melanocytes are not present in the small bowel, the first theory is supported by their presence in the alimentary tract. Another theory suggests melanoma may arise from Schwann cells in the small intestine but remains unverified. According to the third theory, neural crest could be the origin of malignant melanoma. “These potential cells migrate via the umbilical-mesenteric canal and later differentiate into specialized cells, i.e., amine precursors uptake and decarboxylation (APUD) cells, which undergo neoplastic transformation” (9). Based on APUD theory, the ileum (as the distal part of the umbilical-mesenteric canal) should be the most common site of the primary malignant melanoma of the small bowel; however, some authors do not accept the existence of primary melanoma in the small intestine, arguing that primary mucocutaneous melanomas can regress before metastasis or maybe they are too tiny to be detected by clinical investigations (10). Distinguishing a primary from a metastatic intestinal melanoma is a challenging task that may lead to controversies (10). Due to tendency for a more aggressive growth, primary small intestinal melanoma is associated with a worse prognosis (6). Some gastrointestinal melanomas remain with unknown primary sources, even after a thorough examination (11). Based on the research conducted by Sacks and his team, three diagnostic criteria would help to determine the primary type of small intestinal melanoma. These are as follow: 1) the presence of a single lesion, 2) the absence of primary lesions in other organs and no enlargement of draining lymph nodes, and 3) a survival time of over one year after diagnosis (9). Kawashima A. *et al.* reported that most of the patients with gastrointestinal malignant melanoma have clinical presentations like abdominal pain, GI bleeding, and symptoms of obstruction, and about one-third of them are asymptomatic (12).

Histopathological analysis and immunohis-tochemical studies are essential for diagnosis, especially in ruling out mimics. A reliable diagnosis of primary gastrointestinal melanoma requires ruling out metastasis from more common primary sources. In our case with clinical presentation of progressive abdominal pain and peritonitis, there was a single lesion without a history of primary lesion in the skin or mucosal surfaces. The histological study with a concordant immunohistochemical profile was consistent with malignant melanoma. Therefore, a diagnosis of primary malignant melanoma of the small intestine was established. In a follow-up of the patient for six months after surgery, no evidence of recurrence was noted. 

## Conclusion

None.

## Funding

None.

## Conflict of Interest

The authors declare no conflict of interest.
